# Subjective quality of multiple choice questions used in undergraduate courses in orthopedics and other specialties

**DOI:** 10.12669/pjms.36.7.2864

**Published:** 2020

**Authors:** Amina Husnain, Asif Khan, Muhammad Umar Khan, Faisal Nazeer Hussain

**Affiliations:** 1 Amina Husnain, FCPS. Assoc. Prof. Medical Unit-I, Services Institute of Medical Sciences, Lahore, Pakistan; 2 Asif Khan, MS Orth. Senior Registrar Orthopedics, Avicenna Medical College, Lahore, Pakistan; 3 Muhammad Umar Khan, Trainee Resident Orthopedics, Avicenna Medical College, Lahore, Pakistan; 4 Faisal Nazeer Hussain, FCPS Orth. Professor of Orthopedics, Avicenna Medical College, Lahore, Pakistan

**Keywords:** MCQ, Quality, Construct, Assessment

## Abstract

**Background and Objective:**

Multiple Choice Questions (MCQs) can sample broad domains of knowledge efficiently and reliably. The MCQs of lower order C1(Cognitive Level1=Recall of knowledge) do not fulfill this purpose and those of higher cognitive order C2 (Cognitive Level2=Interpret) &C3(Cognitive Level3=Analyze) are better at assessing the problem solving capabilities of the student. Every good educational activity must be supported by quality examination to complete the objectives of a curriculum. The objective of the study was to evaluate MCQs presently being used in internal examinations of medical colleges in Lahore.

**Methods:**

Papers consisting of MCQs from Orthopedics other specialties were collected in June 2019 from different medical colleges of Lahore and reviewed by a senior medical teacher without blinding and without his knowing the scores the students had been awarded before. Question statement, clinical scenarios, options and other mistakes were assessed in each item on predetermined criteria. Cognitive level of the item was determined if it was asking for a recall/identify/ analyze response. The results were tabulated and compared in two groups i.e. Miscellaneous and Orthopedics.

**Results:**

Most of the items(total=589) in both groups were of C1 cognitive level though Orthopedics (229) were slightly better (χ2 = 49.882 P-Value = 0.000 (Statistically Significant). Miscellaneous group (360) was better in quality in making clinical scenarios (χ2 = 29.952 P-Value = 0.000 (Statistically Significant) and writing a question statement without confusion. Options were better written in both groups. A good percentage of items needed to be corrected for mistakes in spellings, grammar and segregation into under graduate level.

**Conclusions:**

The cognitive level of assessment tool s MCQs is quiet low in both groups especially clinical scenario construction can be improved. Mistakes in spellings, grammar and conceptual mediocrity is common in both groups.

## INTRODUCTION

Most medical colleges teach both the undergraduate & the postgraduate. Objective or multiple choice item based papers are an integral part of summative and formative assessments world over^1^. Multiple choice question are being used regularly in various forms e.g “true/false” or “single best-answer” with the intention to assess knowledge. MCQs can sample broad domains of knowledge efficiently and reliably.^1^,^2^ MCQs have traditionally been blamed for being poor in validity while advocates say that they are more reliable. Critics say MCQs promote factual recall and appreciation of isolated facts. But if the MCQs are carefully made, the single best answer MCQs may also test higher-order thinking skills.^1^,^2^ Students are quick to learn from the assessments methods and adapt their learning techniques to pass the next examination which becomes more obvious if the curriculum and assessment are misaligned. Item Writing Flaws are frequently encountered during the (PreHoc) before actual use of the item in real examinations). Next review is done after (Post Hoc) their use. Most experts explain item making flaws and inadequacies in construct occur due to our deviation from the accepted guidelines of making MCQs. Inappropriately worded items, philosophically unchallenging scenarios, lower in cognitive level clinical problems and those soliciting recall will affect the performance of the students. A poorly written scenario may result in a recall rather than the intended analyze or interpretation response. Christian et al cite a study reporting that more than 90% of MCQs in an internal examination were of low cognitive levels and that 46.2% of these MCQs had item writing flaws in them. Coincidently the lower the cognitive level more frequent were the item writing flaws.^2^-^4^ Baig et al in their study in 2014 while evaluating basic sciences examination items in Pakistan reported that the cognitive level of most of the SEQs (83.33%) and MCQs (60%) were at C1-recall level, respectively, and 69 Item Writing Flaws (46%) were found in 150 MCQs.^4^ A study by Naeem et al., (2012) at Aga Khan University (AKU) and Baig et al (2016) agree that any betterment in item quality is bound to faculty development.^1^,^4^,^5^ Another study reported 17% change in the quality of MCQs after attending a short training session about the construction of MCQS.^3^-^5^ Each teaching activity is planned such as to modify cognitive abilities of learners so that they can analyze the clinical problems, solve them, think critically and interpret findings. They can only be made to do this successfully if the assessment does no solicit recall and factual knowledge.^1^,^6^ Educationists insist that the assessment methods should be made known to the students beforehand which has important bearing upon their learning practices and preparation for examinations.^7^ All examinations should be followed by a review later so that learning can be further improved.

The failure of the final outcome of a teaching activity culminates in shaping the final product i.e. the physicians having inadequate competencies and loss of patient care.^7^,^8^ The scenario presented before the stem should provoke an analytic response leading to problem solving. The MCQs of lower order C1 do not fulfill this purpose and C2&C3 are better at assessing the problem solving capabilities of the student. A good educational activity must be supported by an equally purposeful high quality examination to complete the objectives of a curriculum. This necessitates an evaluation of the examination material regularly which became the reason for our present study.

## METHODS

Papers consisting of MCQs from Orthopedics and other Specialties (Miscellaneous group) like medicine, surgery, ENT and urology were collected in June 2019 from different medical colleges of Lahore and reviewed by a senior medical teacher without blinding and without knowing the scores the students had been awarded before. All these had been used a least once in the internal examinations of the final year class. No student results were recalled from college records and each item was analyzed by the same senior medical teacher. Each MCQ item contained a stem and five options. A true response to an item was awarded one mark, while an incorrect response would result in the no deduction. The problem stated in the item was assessed to see if the student would respond by recalling book knowledge as taken as C1. A clinical problem leading to identifying a problem or needing further investigation through more modalities was labeled as C2. C3 was taken as for those items where the question needed a management response. All those scenarios where the diagnosis was very straight forward like a book picture( where reading the data would lead to a single classical conclusion e.g. pain right iliac fossa along with suggestive findings takes one to acute appendicitis) was taken as C1. The question was reviewed for being clear in content and intent i.e. the question or the clinical problem should lead to one option more than the others. Those which seemed less focused or vague in nature were segregated in groups. Each item was analyzed for spelling, grammatical, structural deficiencies like absence of a question statement and those beginning with an Arabic number instead of words were noted separately. The level of the question being suitable to be used in undergraduate examinations.

Those thought to be above the level expected of an undergraduate were marked as Post graduate question. Scenario given in the question was evaluated to be Focused-0, Unfocused can lead to more than one similar options-1, Vague Description-2, Logical clues-3, Data not in sequence/unnecessary information-4. Options were evaluated as being close to the true answer or for being confusing such as “none of the above or all of the above” was taken as a major fault. They were assigned as score: No Fault-0, Irrelevant-1, Implausible-2, Except/All/None of the above-3 and Unfocused-4.

## RESULTS

In all 360 items for Miscellaneous group were included and 229 for Orthopedics were included. All items were systematically evaluated as per predetermined criteria. Cognitive level of the items was found to be C1 in 187/360 (51.94%) in Miscellaneous Group, 52/229 (22.7%) in Orthopedics and 239/589(40.57%) when seen as a combined group Table-I. C2 questions were 111/360(30.83%) in Miscellaneous, 109/229(47.6%) Orthopedics and 220/589(37.36%) in Combined Group. Miscellaneous group was better in cognitive quality of the items (χ2 = 49.882 P-Value = 0.000 (Statistically Significant). Overall χ2 = 49.882 P-Value = 0.000 (Statistically Significant) χ2 = 49.882 P-Value = 0.000 (Statistically Significant)χ2 = 49.882 P-Value = 0.000 (Statistically Significant).

**Table-I:** Cognitive level of Multiple Choice Questions Comparing Orthopedics items to Miscellaneous papers.

Cognitive Levels	Orthopedics	Miscellaneous	Combined
C1 Identify	52 (8.8%)	187 (31.7%)	239 (40.6%)
C2 Interpret	109 (18.5%)	111 (18.8%)	220 (37.4%)
C3 Analyze	68 (11.5%)	62 (10.5%)	130 (22.1%)

Total	229 (38.9%)	360 (61.1%)	589 (100.0%)

χ2 = 49.882 P-Value = 0.000 (Statistically Significant)

When quality of question statement was assessed 350/360(97.22%) were found to be focused in Miscellaneous group, while 195/229(85.2%) in Orthopedics while the combined effect was 545/589(92.52%)Table2(χ2 = 29.952 P-Value = 0.000 (Statistically Significant). Language mistakes and other problems were seen in 97/360 in Miscellaneous group, 55/229 in Orthopedics and 152/589(25.8%) when combined together Table-III (χ2 = 73.237 P-Value = 0.000 (Statistically Significant).

**Table-II:** Quality of Question Statements in MCQ Items Comparison of Orthopedics against Miscellaneous papers.

Quality of Q- Statements	Orthopedics (No)	Miscellaneous (No)	Combined
Focused	195 (33.1%)	350 (59.4%)	545 (92.5%)
Unfocused	32 (5.4%)	10 (1.7%)	42 (7.1%)
Vague Description	2 (0.3%)	0 (0.0%)	0 (0.3%)

Total	229 (38.9%)	360 (61.1%)	589 (100.0%)

χ2 = 29.952 P-Value = 0.000 (Statistically Significant)

**Table-III:** Comparison of Lingual/technical Mistakes in MCQs between Orthopedics & Miscellaneous Items.

Lingual/technical Mistakes	Orthopedics (55 out of 229)	Miscellaneous (97 out of 360)	Combined (152 out of 589)
E-spellings +punctuation	23 (15.1%)	22 (14.5%)	45(29.6%)
EL- E plus grammatical mistakes	3 (2.0%)	0 (0.0%)	3(2.0%)
N-Insufficient data/needs review	1 (0.7%)	5 (3.3%)	6(3.9%)
B-Badly phrased Question	0 (0.0%)	2 (1.3%)	2(1.3%)
V-Very Bad Question	2 (1.3%)	1 (0.7%)	3(2.0%)
R-Review Needed	1 (0.7%)	4 (2.6%)	5(3.3%)
W-Wrong Option	1 (0.7%)	0 (0.0%)	1 (0.7%)
PGL-Post Graduate Level	15 (9.9%)	0 (0.0%)	15 (9.9%)
LQ-Low Quality	2 (1.3%)	0 (0.0%)	2 (1.3%)
S-Scenario not Needed	0 (0.0%)	13 (8.6%)	13 (8.6%)
BN-Begins with number	2 (1.3%)	5 (3.3%)	7(4.6%)
NQ-No Question Statement given	0 (0.0%)	6 (3.9%)	6 (3.9%)
NS-No Scenario given	0 (0.0%)	30 (19.7%)	30 (19.7%)
R-Repeated	5(3.3%)	9 (5.9%)	14(9.2%)

Total	55 (%) (36.2%)	97 (%) (63.8%)	152 (100.0%)

χ2 = 73.237 P-Value = 0.000 (Statistically Significant)

Options in an MCQ form the most important part where distractors are added to provide a challenge. Most of the items in both groups showed that majority had no problem e.g. 310/360(86.11%) in Miscellaneous group and 197/229(86%) in Orthopedics group Table-IV (χ2 = 4.954 P-Value = 0.292 (Statistically Non-Significant).

**Table-IV:** Comparison of Quality of Options statements in Items of MCQs in Orthopedics & Miscellaneous papers.

Quality of Q- Statements	Orthopedics (No)	Miscellaneous (No)	Combined
No Fault	197 (33.4%)	310 (52.6%)	507 (86.1%)
Irrelevant	3 (0.5%)	2 (0.3%)	5 (0.8%)
Implausible	8 (1.4%)	5 (0.8%)	13 (2.2%)
Except/All/None of the above	14 (2.4%)	26 (4.4%)	40 (6.8%)
Unfocused	7 (1.2%)	17 (2.9%)	24 (4.1%)

Total	229 (38.9%)	360 (61.1%)	589 (100.0%)

χ2 = 4.954 P-Value = 0.292 (Statistically Non-Significant)

Clinical scenarios are added to the item to see the analytical and applied thinking of the student. In both the groups majority of the scenarios were good quality while Miscellaneous group 347/360(96.38%) prevailed upon the Orthopedics 184/22(80.3%) Table-V (χ2 = 47.071 P-Value = 0.000 (Statistically Significant).

**Table-V:** Comparison of Quality of Clinical Scenario in SAQs of Miscellaneous and Orthopedics Groups.

Quality of Clinical Scenario	Orthopedics (No)	Miscellaneous (No)	Combined
Focused	184 (31.2%)	347 (58.9%)	531 (90.2%)
Unfocused can lead to more than one similar options	24 (4.1%)	2 (0.3%)	26 (4.4%)
Vague Description	13 (2.2%)	9 (1.5%)	22 (3.7%)
Logical clues	7 (1.2%)	1 (0.2%)	8 (1.4%)
Data not in sequence/unnecessary information	1 (0.2%)	1 (0.2%)	2 (0.3%)

Total	229 (38.9%)	360 (61.1%)	589 (100.0%)

χ2 = 47.071 P-Value = 0.000 (Statistically Significant)

## DISCUSSION

Every teaching program depends highly upon the alignment of the assessment with the objectives of the curriculum. No learning outcome can be achieved until assessment and evaluation being done during the program is scientific and proactive (according to a pre-laid blue print). In an MCQ item the correct options should be defensibly correct and distracters should be defensibly incorrect. Multiple true and false option MCQs have a disadvantage over single best choice MCQs that they are more complicated in scoring and more difficult to make.^9^ All paper setting faculty members should be trained to follow the blue print laid down earlier.^5^ Only then can we improve the cognitive level of the MCQs and reduce the IWFs. Single best choice MCQs are preferred because they are easy to answer, convenient to conduct for teachers and they are versatile in use. But they are difficult to make in a quality manner.^9^ Our study has found that Miscellaneous groups had more (51.94%) MCQs in C1 group than Orthopedics (22.7%) Table-I which points out more experience of the faculty at making examination material. Without active monitoring of the teaching/assessment methodology, the student will change their strategy to rote learning rather than develop an analytical approach to get a deeper grasp of the subject.^7^ Faculty specialization has also led to a poorly balanced curriculum as each medical unit is now being occupied by super specialists like endocrinology or gastroenterologists who have lesser and lesser experience of teaching medicine.^8^ Modern undergraduate curricula tend to include only basic information regarding subspecialties hence the item writing examiner has to be very careful so that one does not cross over to the post graduate level making the examination beyond the scope of his students e.g. the students are given very basic insight in the bone tumors and it becomes very difficult for the student if he is dragged into details and differential diagnosis of bone malignancies. Table-II shows a similar picture where overall assessment of the question statement shows that Miscellaneous group shows 97.22% questions to be more focused and Orthopedics group items were 85.2% adequately focused and rest were vague and confusing. Orthopedics department has to arrange less tests than medicine and surgery where the curriculum is bigger in size. When the items were analyzed for spelling mistakes and grammatical short comings in Miscellaneous group and in Orthopedics showed faults e.g. E-spellings +punctuation, EL- E plus grammatical mistakes, N-Insufficient data/needs review, B-Badly phrased Question, V-Very Bad Question, R-Review Needed, W-Wrong Option etc. shown in Table-III. These criteria have been suggested by us and are being used first time. It would need to stand test of time upon review periodically. When options in the items were assessed they were found to be of good quality in both groups Table-IV. Clinical Scenario building lies at the heart of any question may it be an MCQ or SAQ. Orthopedic MCQs showed much lower quality than the other group 184/229 (80.3%) vs 347/360 (96.38%) and when put together 531/589 (90.15%) Table-V. It has been pointed out by William G. Rothstein (1987) that over specialization(e.g. a surgeon from bariatric surgery unit teaching general surgery) of faculty leads “to loss of responsibility in the faculty who then lose responsibility of the undergraduate students”.^8^ Probably a capacity building after a need based analysis for Orthopedics maybe of help. Written assessments are practical, though they are commonly used yet they have their own disadvantages: they are difficult to construct flawlessly—the statements have to be defensibly true or absolutely false.^10^ The whole purpose of teaching fails if required skills are not learnt and the capacity to analyze a clinical problem is not acquired. Our endevour has been focused to see the quality of initial construct(pre-hoc) of the Multiple choice question items because the prevalent practices tend to depend upon statistical evaluation like calculating validity and discrimination indices(pos-hoc). There is another objection to use of MCQs is that when a student chooses false as response we conclude that he known that this is not the answer but it still remains unknown whether he knows the right answer.^10^ Assessment plays as important role in the qualities of the future doctor as it shapes the interest and learning strategies of the medical students. Written examinations involving multiple-choice questions, case-based questions, and essays type questions have an accepted face and content validity; but the association between students’ performance in the assessment and performance in real life situations is unkown especially when it comes to non scholastic learning^10^,^11^ An over simplistic recall soliciting question will only result in rote learning. The reason can be a dominant overlay of attitude and non-scholastic learning over the theoretical teaching at undergraduate levels.^11^

### Limitations of the study

Our study does not calculate discrimination and difficulty indices as is generally recommended for MCQs. Our Study is a very small scale exploratory effort that is trying to identify the standard/faults/possibilities of improvements at undergraduate level. Its best contribution is that it points to analyze the situation in a scientific manner. Therefore, our observations should not be generalized until a more scientific large scale study has been done.

## CONCLUSION

The cognitive level of assessment tools MCQs used in the internal examinations in Lahore is quiet low especially although specialties other than Orthopedics are better in quality. Clinical scenario construction can be improved. Mistakes in spellings, grammar are frequent and structural mediocrity is common especially in the options given. Need based analysis of the situation should be done to point out groups of teachers who should be trained and post use review of all items should be made regularly.

### Author’s Contribution:

**AH:** Preparation/final approval of manuscript,

**FNH:** Conceived the idea/final approval of manuscript.

**AK & MUK:** Data Collection and preparation of manuscript.
